# Morphometric study of the neural ossification centers of the atlas and axis in the human fetus

**DOI:** 10.1007/s00276-016-1681-2

**Published:** 2016-05-03

**Authors:** Mariusz Baumgart, Marcin Wiśniewski, Magdalena Grzonkowska, Bogdan Małkowski, Mateusz Badura, Michał Szpinda

**Affiliations:** 1 Department of Normal Anatomy, The Ludwik Rydygier Collegium Medicum in Bydgoszcz, The Nicolaus Copernicus University in Toruń, Łukasiewicza 1 Street, 85-821 Bydgoszcz, Poland; 2 Department of Positron Emission Tomography and Molecular Imaging, The Ludwik Rydygier Collegium Medicum in Bydgoszcz, The Nicolaus Copernicus University in Toruń, Łukasiewicza 1 Street, 85-821 Bydgoszcz, Poland

**Keywords:** Neural, Ossification center, Atlas, Axis, Size, Growth dynamics, Human fetus, Regression analysis

## Abstract

**Purposes:**

The knowledge of the developing cervical spine and its individual vertebrae, including their neural processes may be useful in the diagnostics of congenital vertebral malformations. This study was performed to quantitatively examine the neural ossification centers of the atlas and axis with respect to their linear, planar and volumetric parameters.

**Methods:**

Using the methods of CT, digital-image analysis and statistics, the size of neural ossification centers in the atlas and axis in 55 spontaneously aborted human fetuses aged 17–30 weeks was studied.

**Results:**

Without any male–female and right–left significant differences, the best fit growth dynamics for the neural ossification centers of the atlas and axis were, respectively, modelled by the following functions: for length: *y* = −13.461 + 6.140 × ln(age) ± 0.570 and *y* = −15.683 + 6.882 × ln(age) ± 0.503, for width: *y* = −4.006 + 1.930 × ln(age) ± 0.178 and *y* = −3.054 + 1.648 × ln(age) ± 0.178, for cross-sectional area: *y* = −7.362 + 0.780 × age ± 1.700 and *y* = −9.930 + 0.869 × age ± 1.911, and for volume: *y* = −6.417 + 0.836 × age ± 1.924 and *y* = −11.592 + 1.087 × age ± 2.509.

**Conclusions:**

The size of neural ossification centers of the atlas and axis shows neither sexual nor bilateral differences. The neural ossification centers of the atlas and axis grow logarithmically in both length and width and linearly in both cross-sectional area and volume. The numerical data relating to the size of neural ossification centers of the atlas and axis derived from the CT and digital-image analysis are considered specific-age reference values of potential relevance in both the ultrasound monitoring and the early detection of spinal abnormalities relating to the neural processes of the first two cervical vertebrae in the fetus.

## Introduction

A review of the literature in relation to the spinal ossification centers has displayed various methods used for their assessment: from histologic through radiographic to modern imaging methods, such as ultrafast NMR [5] and 3D ultrasound [21, 31]. The ossification process in cervical vertebrae is quite intricate and discussable [5, 11, 12, 14–16]. There are three ossification centers per vertebra within cervical vertebrae C3–C7, one in its body and one in either neural arch. The first two cervical vertebrae are atypical, and their development significantly differs from that of the other cervical vertebrae [5, 11, 15, 16]. In the atlas, the three ossification centers occur: one located in its anterior arch and two located in its posterior arch. On the other hand, the four ossification centers are observed in the axis: one in its body, one in its dens and one in either neural process.

Of note, a high-resolution ultrasound permits a precise in utero evaluation of ossification centers, thus enabling both the monitoring of fetal development and the early detection of skeletal malformations. The understanding of the evolution of cervical vertebrae, including their neural processes in particular, may be useful in the diagnostics of congenital abnormalities, such as spina bifida, achondrogenesis, and skeletal dysplasias [26, 27, 30, 31]. Accurate data on the ossification of cervical vertebrae are also useful in forensics and archeology [14, 15].

As reported, the very first ossification centers in the spine appear at week 8 in the neural processes of upper cervical vertebrae, i.e., the atlas and axis, and consecutively progress caudad [11, 12]. To date, however, the evolution of linear, planar and spatial dimensions of the neural ossification centers has been established in detail using computed tomography and digital image analysis only for three typical vertebrae: C4 [4], T6 [28] and L3 [29]. Therefore, in the present study, we focused on the advanced morphometric analysis of the neural ossification centers of the atlas and axis.

The aims of the study were:

- to quantitatively analyze the neural ossification centers in the first two cervical vertebrae with respect to their linear, planar and volumetric parameters so as to determine their age-specific reference values,
- to examine the possible sexual differences regarding the analyzed parameters, and
- to compute growth dynamics for the analyzed parameters, including best-matched mathematical models.

## Materials and methods

The study material comprised 55 human fetuses of both sexes (27 males and 28 females) aged 17–30 weeks of gestation, originating from spontaneous abortions and preterm deliveries. The material was acquired before the year 2000 and remains part of the specimen collection of our Department of Normal Anatomy. The experiment was sanctioned by the Bioethics Committee of the University (approval KB 275/2011). The fetal age was determined based on the crown-rump length. Table 1 lists the characteristics of the study group, including age, number and sex of the fetuses.

**Table 1:** Age, number and sex of the fetuses studied

Gestational age	Crown-rump length (mm)				Number of fetuses	Sex	
Weeks (Hbd-life)	Mean	SD	Min.	Max.		♂	♀
17	115.00	–	115.00	115.00	1	0	1
18	133.33	5.77	130.00	140.00	3	1	2
19	149.50	3.82	143.00	154.00	8	3	5
20	161.00	2.71	159.00	165.00	4	2	2
21	174.75	2.87	171.00	178.00	4	3	1
22	185.00	1.41	183.00	186.00	4	1	3
23	197.60	2.61	195.00	202.00	5	2	3
24	208.67	3.81	204.00	213.00	9	5	4
25	214.00	–	214.00	214.00	1	0	1
26	229.00	5.66	225.00	233.00	2	1	1
27	237.50	3.33	233.00	241.00	6	6	0
28	249.50	0.71	249.00	250.00	2	0	2
29	253.00	0.00	253.00	253.00	2	0	2
30	263.25	1.26	262.00	265.00	4	3	1
Total					55	27	28

**Fig. 1:**
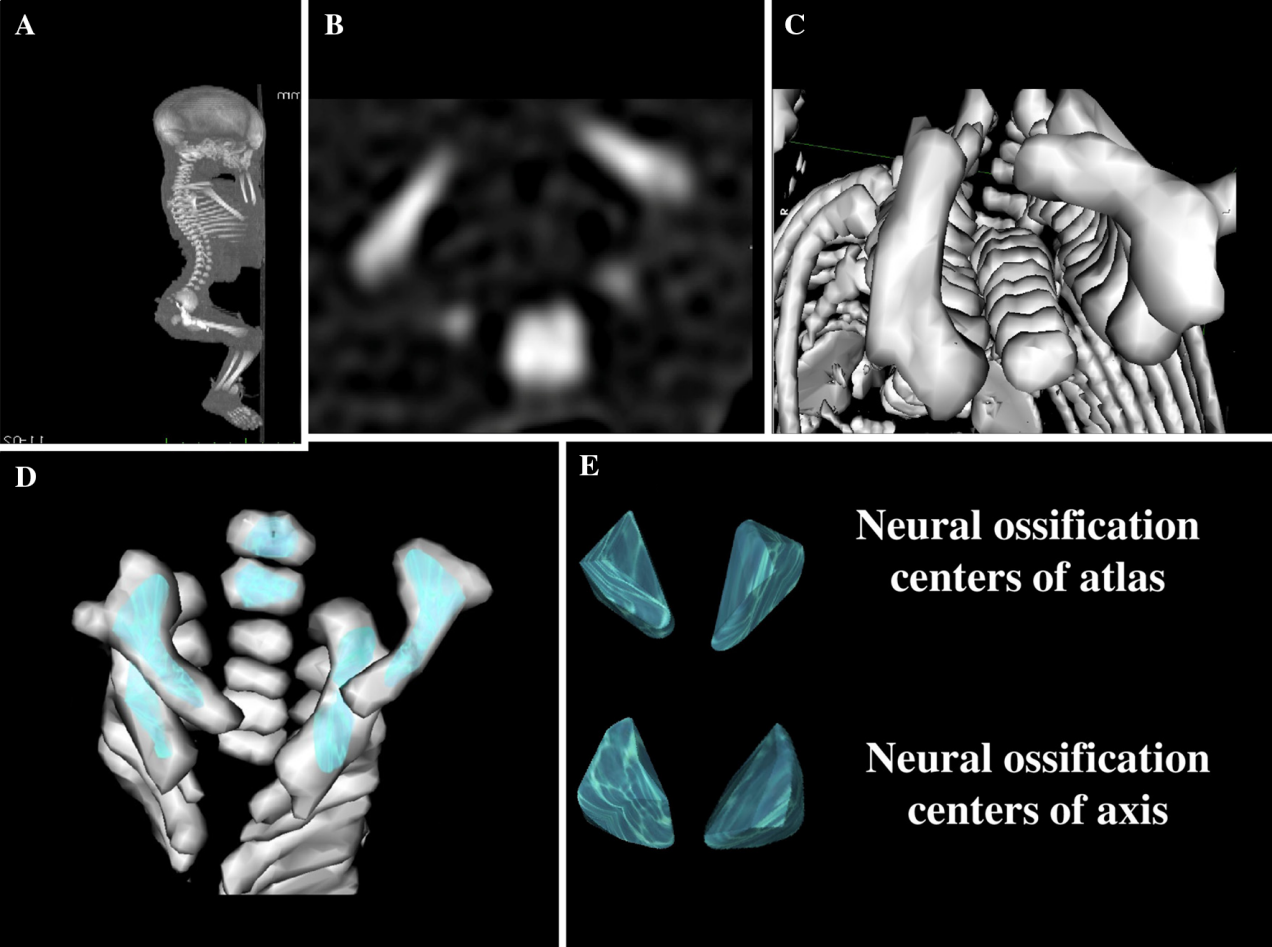
CT of a male fetus aged 22 weeks in the sagittal (**a**) and transverse (**b**) projections of the cervical vertebrae, reconstruction of the cervical vertebrae in the transverse projection (**c**), reconstruction of the atlas and axis using Osirix 3.9 (**d**), and neural ossification centers of the atlas and axis (**e**)

Using Siemens Biograph 128 mCT, fetal CT scans were recorded in DICOM formats with the reconstructed slice width option of 0.4 mm (Fig. 1a). Such a technique is a prerequisite for further spatial reconstructions (Fig. 1b–e) and morphometric analysis of objects given [6, 8]. The gray scale in Hounsfield units of achieved CT pictures ranged from −275 to −134 for a minimum, and from +1165 to +1558 for a maximum. Thus, the window width (WW) alternated from 1404 to 1692, and the window level (WL) varied from +463 to +712. Osirix 3.9 allows us to carry out any type of linear, planar and 3D reconstructions of the studied objects along with their precise quantitative analysis (Figs. 1 and 3).

**Fig. 2:**
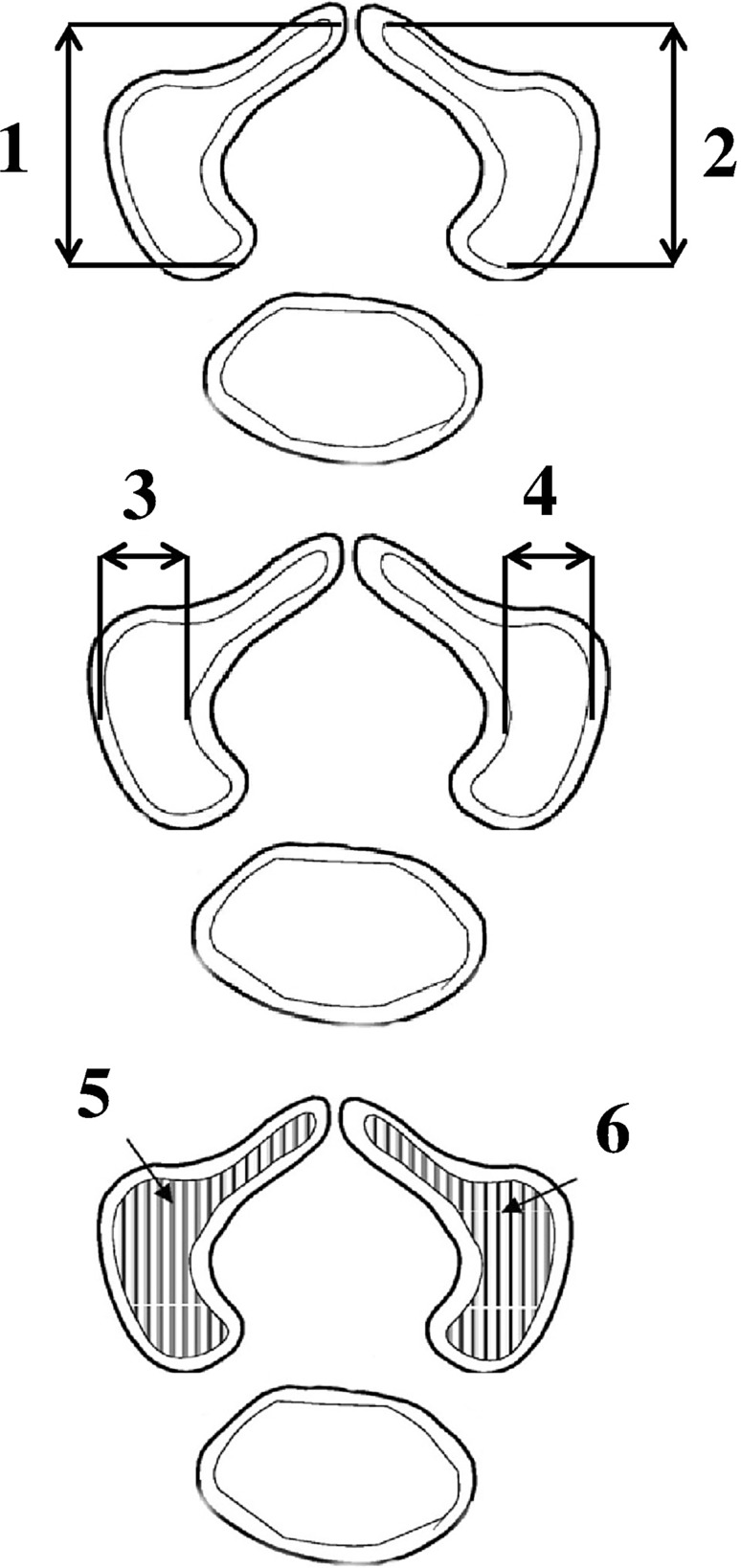
Diagram showing the measurements of the neural ossification centers of the atlas and axis
- 3, 4—width of the ossification centers in the left and right neural processes (in mm), based on the distance between their medial and lateral borderlines in the transverse plane (Fig. 2),
- 5, 6—cross-sectional area of the ossification centers in the left and right neural processes (in mm^2^), based on their determined contours in the transverse plane (Fig. 2),
- 7, 8—volume of the ossification centers in the left and right neural processes (in mm^3^), based on the advanced spatial reconstructions of objects (Figs. 3 and 4).

For each neural process of the atlas and axis, a quantitative assessment of the following eight parameters was conducted:

- 1, 2—length of the ossification centers in the left and right neural processes (in mm), based on the distance between their anterior and posterior borderlines in the transverse plane (Fig. 2),

**Fig. 3:**
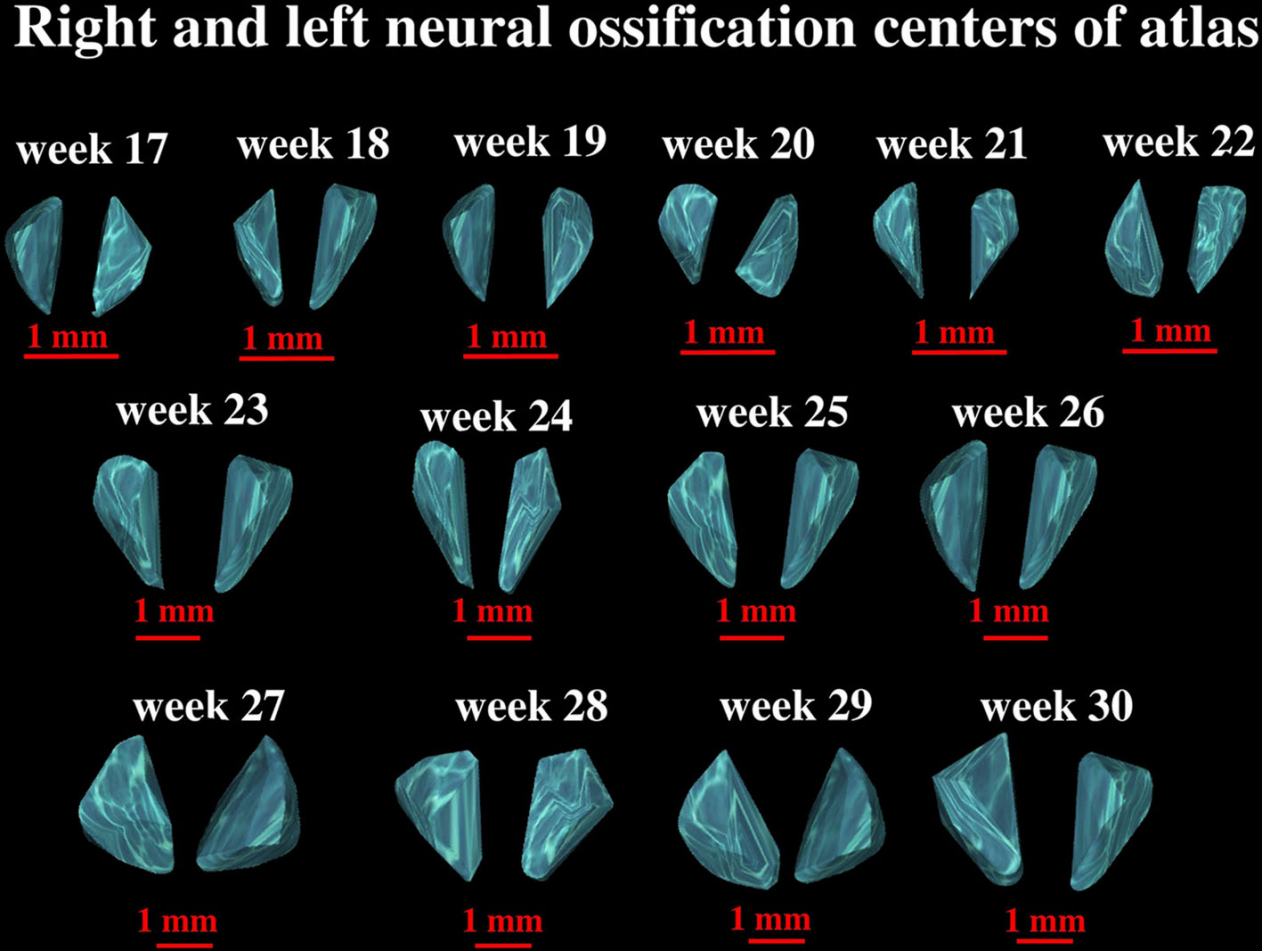
3D reconstruction of the neural ossification centers of the atlas in fetuses aged 17–30 weeks, assessed by Osirix 3.9

**Fig. 4:**
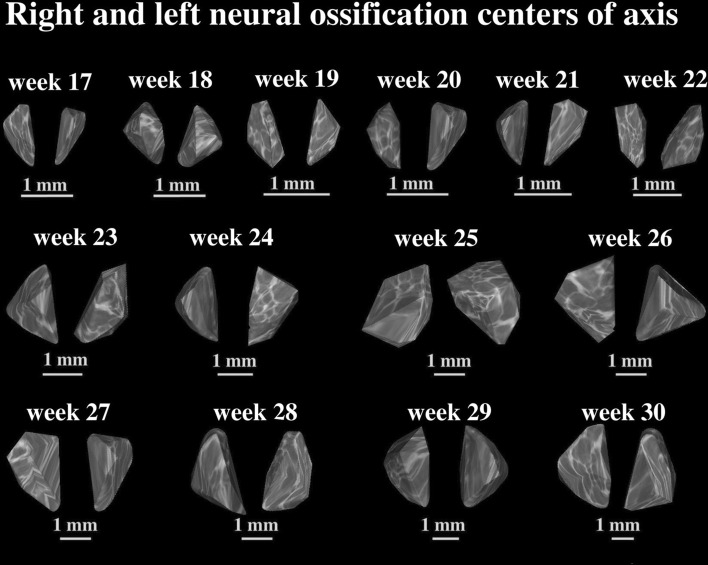
3D reconstruction of the neural ossification centers of the axis in fetuses aged 17–30 weeks, assessed by Osirix 3.9

The numerical data were statistically analyzed. The distribution of variables was checked using the Shapiro–Wilk (W) test, while the homogeneity of variance was checked using the Fisher test. The results are expressed as arithmetic means with the standard deviation (SD). To compare the means, Student’s *t* test for independent variables and one-way analysis of variance were used. The Tukey test was used for the post hoc analysis. If no similarity of variance occurred, the non-parametric Kruskal–Wallis test was used. To check any differences between male and female fetuses, they were separated into the following five age groups: 17–19, 20–22, 23–25, 26–28 and 29–30 weeks of gestation. The characterization of developmental dynamics of the analyzed parameters was based on the linear and curvilinear regression analysis. The match between the estimated curves and the numerical data was evaluated based on the coefficient of determination (*R* ^2^).

## Results

The statistical analysis revealed neither sexual nor bilateral differences regarding all analyzed parameters. Therefore, we investigated the developmental dynamics of the established parameters without taking sex or side of the body into account.

The mean values of the length, width, cross-sectional area and volume of the ossification centers in the right and left neural processes have been given separately for the atlas (Table 2) and axis (Table 3).

**Table 2:** Morphometric parameters of the neural ossification centers of atlas

Gestational age (weeks)	*n*	Ossification centers of neural processes of atlas															
		Length (mm)				Width (mm)				Cross-sectional area (mm^2^)				Volume (mm^3^)			
		Right		Left		Right		Left		Right		Left		Right		Left	
		Mean	SD	Mean	SD	Mean	SD	Mean	SD	Mean	SD	Mean	SD	Mean	SD	Mean	SD
17	1	4.57		3.83		1.27		1.43		4.20		6.60		8.42		7.12	
18	3	4.80	0.01	4.08	0.52	1.66	0.16	1.61	0.03	7.80	0.46	7.53	0.15	9.87	0.72	9.46	0.83
19	8	4.83	0.28	4.53	0.29	1.59	0.07	1.63	0.07	6.90	1.60	8.50	1.82	10.68	1.03	8.19	1.48
		↓ (*P* < 0.05)		↓ (*P* < 0.05)		↓ (*P* < 0.01)		↓ (*P* < 0.05)		↓ (*P* < 0.05)		↓ (*P* < 0.05)		↓ (*P* < 0.05)		↓ (*P* < 0.01)	
20	4	4.94	0.48	3.86	0.18	1.76	0.14	1.70	0.10	7.33	1.15	6.88	1.19	10.25	1.77	8.02	0.79
21	4	5.62	0.36	4.98	0.62	1.91	0.02	1.84	0.13	8.25	0.74	8.18	1.05	9.02	0.62	10.73	1.28
22	4	5.60	0.34	5.97	0.60	1.99	0.16	1.95	0.14	10.13	1.21	10.20	0.88	12.20	0.27	11.20	0.42
		↓ (*P* < 0.05)		↓ (*P* < 0.05)		↓ (*P* < 0.01)		↓ (*P* < 0.05)		↓ (*P* < 0.001)		↓ (*P* < 0.05)		↓ (*P* < 0.01)		↓ (*P* < 0.01)	
23	5	5.89	0.72	5.87	0.80	2.04	0.23	2.09	0.13	11.42	3.23	11.18	2.07	13.70	1.99	12.86	2.84
24	9	5.74	0.46	5.65	0.68	2.31	0.22	2.10	0.11	11.51	2.35	11.09	1.37	15.64	0.72	13.27	1.90
25	1	6.46		5.79		2.00		2.12		10.20		10.30		14.00		12.70	
		↓ (*P* < 0.01)		↓ (*P* < 0.01)		↓ (*P* < 0.05)		↓ (*P* < 0.01)		↓ (*P* < 0.01)		↓ (*P* < 0.01)		↓ (*P* < 0.05)		↓ (*P* < 0.01)	
26	2	6.94	0.41	6.42	0.16	2.53	0.14	2.22	0.06	10.65	0.92	12.55	1.48	15.65	0.64	14.52	3.37
27	6	6.59	0.78	6.39	1.19	2.26	0.19	2.10	0.13	12.92	2.87	13.05	3.22	15.98	3.76	14.60	2.56
28	2	7.21	0.02	7.55	0.42	2.51	0.30	2.20	0.21	13.85	2.19	15.40	0.01	16.80	1.84	16.65	0.21
		↓ (*P* < 0.05)		↓ (*P* < 0.05)		↓ (*P* < 0.05)		↓ (*P* < 0.01)		↓ (*P* < 0.001)		↓ (*P* < 0.05)		↓ (*P* < 0.01)		↓ (*P* < 0.05)	
29	2	7.23	0.01	6.89	0.71	2.85	0.07	2.28	0.01	14.90	0.14	11.80	0.14	17.75	0.07	14.35	0.07
30	4	7.37	0.35	7.68	0.61	2.75	0.21	2.31	0.15	17.80	1.09	15.45	0.81	21.20	0.87	16.21	1.87

**Table 3:** Morphometric parameters of the neural ossification centers of axis

Gestational age (weeks)	*n*	Ossification centers of neural processes of axis															
		Length (mm)				Width (mm)				Cross-sectional area (mm^2^)				Volume (mm^3^)			
		Right		Left		Right		Left		Right		Left		Right		Left	
		Mean	SD	Mean	SD	Mean	SD	Mean	SD	Mean	SD	Mean	SD	Mean	SD	Mean	SD
17	1	4.57		3.83		1.27		1.43		4.60		5.30		8.55		6.36	
18	3	4.80	0.01	4.08	0.52	1.66	0.16	1.61	0.03	6.03	0.55	8.23	1.06	9.38	0.81	8.74	0.91
19	8	4.83	0.28	4.53	0.29	1.59	0.07	1.63	0.07	5.74	0.09	6.63	0.91	8.06	1.24	8.29	0.67
		↓ (*P* < 0.01)		↓ (*P* < 0.01)		↓ (*P* < 0.01)		↓ (*P* < 0.05)		↓ (*P* < 0.01)		↓ (*P* < 0.05)		↓ (*P* < 0.01)		↓ (*P* < 0.001)	
20	4	4.94	0.48	3.86	0.18	1.76	0.14	1.70	0.10	6.90	1.87	6.28	0.46	9.40	2.96	9.21	1.80
21	4	5.62	0.36	4.98	0.62	1.91	0.02	1.84	0.13	8.40	0.80	6.23	0.35	11.18	0.45	11.02	1.97
22	4	5.60	0.34	5.97	0.60	1.99	0.16	1.95	0.14	10.35	1.08	10.13	1.21	12.18	1.01	12.26	1.86
		↓ (*P* < 0.05)		↓ (*P* < 0.05)		↓ (*P* < 0.05)		↓ (*P* < 0.01)		↓ (*P* < 0.01)		↓ (*P* < 0.05)		↓ (*P* < 0.001)		↓ (*P* < 0.05)	
23	5	5.89	0.72	5.87	0.80	2.04	0.23	2.09	0.13	9.90	1.37	9.16	2.31	13.78	2.15	12.58	3.04
24	9	5.74	0.46	5.65	0.68	2.31	0.22	2.10	0.11	12.33	2.12	8.60	2.52	16.38	2.47	13.53	3.69
25	1	6.46		5.79		2.00		2.12		13.80		13.90		19.10		16.90	
		↓ (*P* < 0.05)		↓ (*P* < 0.05)		↓ (*P* < 0.05)		↓ (*P* < 0.05)		↓ (*P* < 0.05)		↓ (*P* < 0.01)		↓ (*P* < 0.05)		↓ (*P* < 0.01)	
26	2	6.94	0.41	6.42	0.16	2.53	0.14	2.22	0.06	13.80	1.13	13.80	3.39	18.90	3.68	17.55	5.73
27	6	6.59	0.78	6.39	1.19	2.26	0.19	2.10	0.13	13.33	2.72	12.42	2.75	17.08	4.00	14.57	4.11
28	2	7.21	0.02	7.55	0.42	2.51	0.30	2.20	0.21	14.05	0.92	15.10	1.56	18.95	1.77	16.95	0.07
		↓ (*P* < 0.05)		↓ (*P* < 0.05)		↓ (*P* < 0.01)		↓ (*P* < 0.05)		↓ (*P* < 0.01)		↓ (*P* < 0.05)		↓ (*P* < 0.01)		↓ (*P* < 0.01)	
29	2	7.23	0.01	6.89	0.71	2.85	0.07	2.28	0.01	16.65	0.07	12.80	0.57	22.50	0.14	21.55	0.21
30	4	7.37	0.35	7.68	0.61	2.75	0.21	2.31	0.15	16.80	0.91	14.90	0.83	21.15	1.97	18.43	0.98

**Fig. 5:**
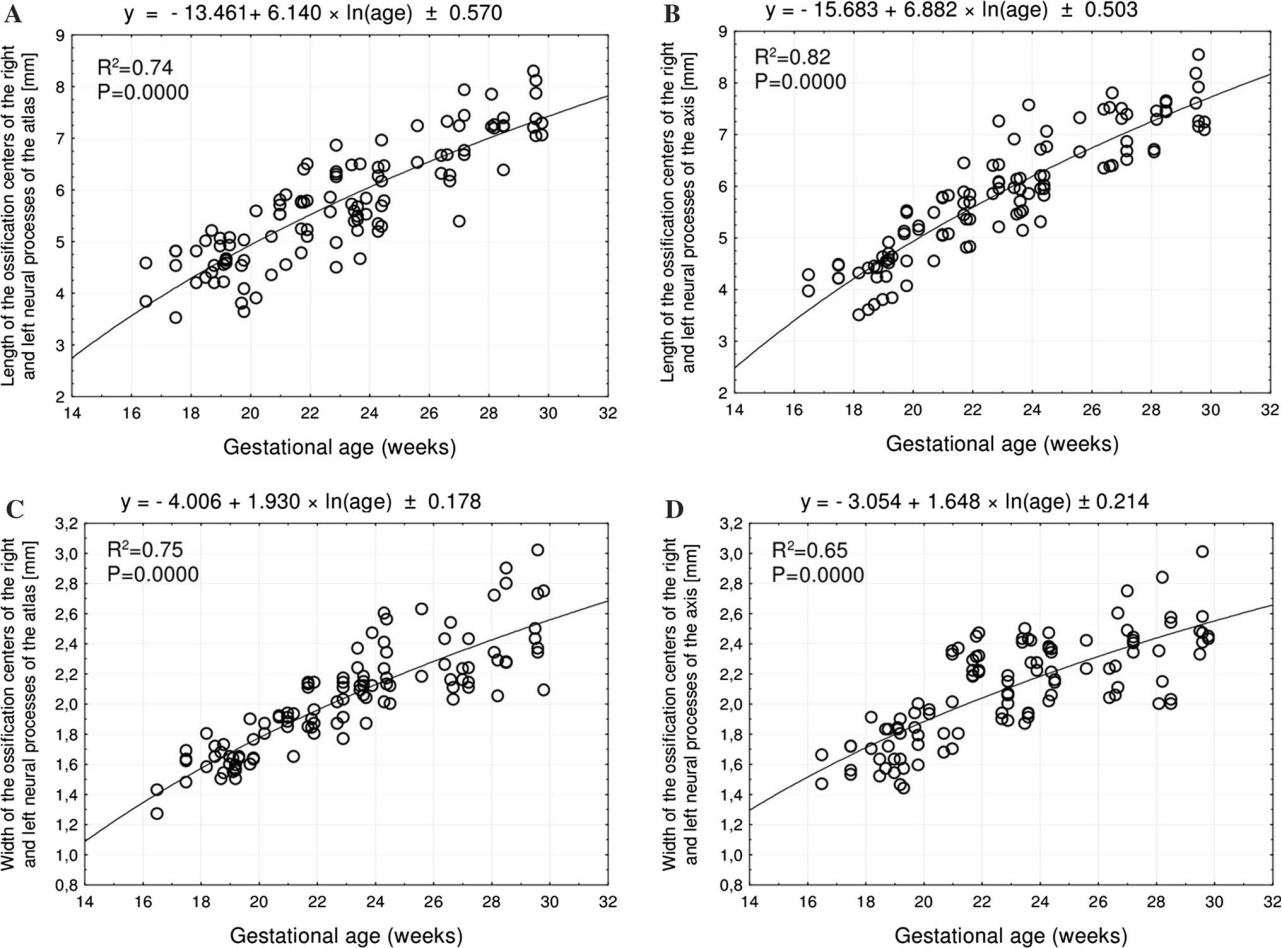
Regression lines for length of the atlantal (**a**) and axial (**b**) neural ossification centers, and for the width of the atlantal (**c**) and axial (**d**) neural ossification centers

The developmental dynamics of length and width of the neural ossification centers in the atlas and axis followed natural logarithmic functions. Between 17 and 30 weeks of gestation, the mean length of the ossification centers in the atlantal neural processes increased from 4.57 to 7.37 ± 0.35 mm on the right, and from 3.83 to 7.68 ± 0.61 mm on the left, following the function *y* = −13.461 + 6.140 × ln(age) ± 0.570 (*R* ^2^ = 0.74) (Fig. 5a). At the same time, the mean length of the ossification centers in the axial neural processes increased from 3.97 to 7.93 ± 0.62 mm 

on the right and from 4.28 to 7.31 ± 0.19 mm on the left, following the function *y* = −15.683 + 6.882 × ln(age) ± 0.503 (*R* ^2^ = 0.82) (Fig. 5b).

Simultaneously, the mean width of the ossification centers in the atlantal neural processes increased from 1.27 to 2.75 ± 0.21 mm on the right, and from 1.43 to 2.31 ± 0.15 mm on the left, following the function *y* = −4.006 + 1.930 × ln(age) ± 0.178 (*R* ^2^ = 0.75) Fig. 5c). The mean width of the ossification centers in the axial neural processes increased from 1.66 to 2.63 ± 0.26 mm on the right and from 1.47 to 2.42 ± 0.06 mm on the left, following the function: *y* = −3.054 + 1.648 × ln(age) ± 0.178 (*R* ^2^ = 0.65) (Fig. 5d).

**Fig. 6:**
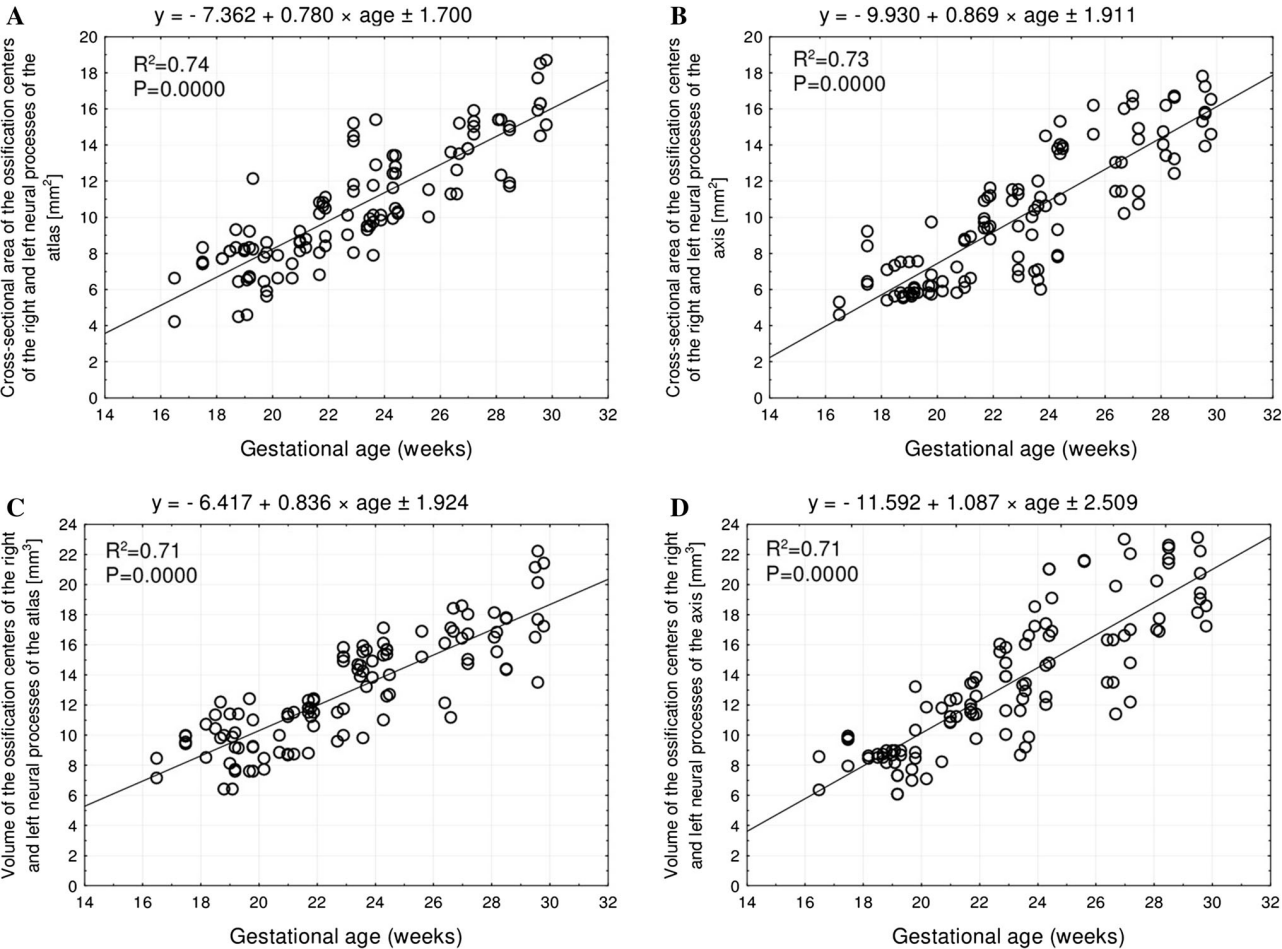
Regression lines for cross-sectional area of the atlantal (**a**) and axial (**b**) neural ossification centers, and for volume of the atlantal (**c**) and axial (**d**) neural ossification centers

In the studied age range, the mean cross-sectional area of the ossification centers in the atlantal neural processes varied between 4.20 and 17.80 ± 1.09 mm^2^ on the right, and between 6.60 and 15.45 ± 0.81 mm^2^ on the left, according to the linear function: *y* = −7.362 + 0.780 × age ± 1.700 (*R* ^2^ = 0.74) (Fig. 6a). The mean cross-sectional area of the ossification centers in the axial neural processes varied between 4.60 and 16.80 ± 0.91 mm^2^ on the right side, and between 5.30 and 14.90 ± 0.83 mm^2^, following the linear function: *y* = −9.930 + 0.869 × age ± 1.911 (*R* ^2^ = 0.73) (Fig. 6b).

The mean volume of the ossification centers in the atlantal neural processes increased from 8.42 to 21.20 ± 0.87 mm^3^ on the right side, and from 7.12 to 16.21 ± 1.87 mm^3^, following the function: *y* = −6.417 + 0.836 × age ± 1.924 (*R* ^2^ = 0.71) (Fig. 6c). The mean volume of the ossification centers in the axial neural processes gained from 8.55 to 21.15 ± 1.97 mm^3^ on the right and from 6.36 to 18.43 ± 0.98 mm^3^, in accordance with the function: *y* = −11.592 + 1.087 × age ± 2.509 (*R* ^2^ = 0.71) (Fig. 6d).

## Discussion

Spinal ossification centers in the bodies and neural arches progress autonomously. The vertebral body ossification centers first appear in the lower thoracic (T10–T12) and upper lumbar (L1–L3) segments [2]. The consecutive ossification process of vertebral bodies simultaneously proceeds both cephalad (in the cranial direction) and caudad (in the caudal direction); the former follows more intensively than the latter [3]. On the other hand, the neural ossification centers initially occur in the atlas at week 8, and hence, the spinal ossification process continues caudad [31]. Szpinda et al. [26] carried out a comprehensive quantitative characterization of the neural ossification centers throughout the fetal spine. As described, a reduction in all measured linear, planar and volumetric parameters in the caudal direction was unveiled. The biggest ossification centers were found in the neural processes of cervical vertebrae, while the smallest ones referred to sacral vertebrae. According to these authors, this must have mirrored the sequence of ossification of neural processes that followed caudad. As reported by Skórzewska et al. [25], in fetuses aged 10–11 weeks, the neural ossification centers existed within all cervical and upper thoracic vertebrae, and since week 12, they have already been presented in the neural processes of lower thoracic and lumbar vertebrae. However, Bagnall et al. [1–3] observed the onset of ossification in the neural processes at the same time in the cervical and thoracolumbar parts, which indubitably contradicted the hypothesis of both cephalad and caudad progressions of ossification in neural processes. Anyway, in every vertebra, its neural processes begin to ossify near the transverse processes, and subsequently continue in a 3D fashion. This permits the growth of the superior and inferior articular processes [9, 31]. The initiation of the ossification process within neural arches may result from movements of the fetus and its specific skeletal muscles [1].

Vignolo et al. [31] stated a relationship between fetal sex and the development of ossification centers, proving the earlier development of female fetuses compared to male fetuses. As a consequence, male fetuses of the same age were more problematic when assessed by ultrasound. Our studies, both the present and previous ones [4, 26–30] did not identify any sexual differences regarding the developmental dynamics of the spinal ossification centers.

Our previous examinations involving human fetuses aged 17–30 weeks presented a comprehensive morphometric analysis of the spine, including growth curves computed for typical vertebrae: C4 [4], T6 [28] and L3 [29]. However, Castellana and Kósa [5] were the only group to quantitatively evaluate the atlantal and axial ossification centers in 106 human fetuses aged 16–40 weeks. The numerical data from the aforementioned papers are essential for this discussion. In the present study, both the length and width of the neural ossification centers in the atlas and axis increased following logarithmic functions. The length of the ossification center of the atlantal neural process increased in accordance with the regression: *y* = −13.461 + 6.140 × ln(age) ± 0.570. As far as the ossification centers of the axial neural processes are concerned, their length increased following the function: *y* = −15.683 + 6.882 × ln(age) ± 0.503. In turn, the width of the neural ossification centers increased logarithmically as follows: *y* = −4.006 + 1.930 × ln(age) ± 0.178 for the atlas, and *y* = −3.054 + 1.648 × ln(age) ± 0.178 for the axis. According to Castellana and Kósa [5], the ossification centers of the atlantal neural arch increased following the function: *y* = 3.834 (neural arch length) − 0.239 (maximum neural arch width) + 0.413 (lamina length) + 2.713 (neural arch transverse dimension) − 2.770 (lamina height) + 2.396 (superior articular surface length) − 4.903 (superior articular surface width) − 2.491 (inferior articular surface length) + 1.190 (inferior articular surface width) + 5.409. Furthermore, the ossification centers of the axial arch increased following the function: *y* = −1.532 (neural arch length) + 6.432 (neural arch transverse dimension) − 0.166 (lamina length) + 2.211 (anterior arch width) − 2.975 (lamina height) − 3.286 (inferior articular surface length) + 8.330 (inferior articular surface width) + 2.125.

Logarithmic increases in both the length and width of the neural ossification centers were also observed for vertebrae C4, T6 and L3. In vertebra C4 on the right and left sides, the length increased with age, following the functions: *y* = −19.601 + 8.018 × ln (age) ± 0.369 and *y* = −15.804 + 6.912 × ln(age) ± 0.471, respectively, while the width increased following the functions: *y* = −5.806 + 2.587 × ln(age) ± 0.146 and *y* = −5.621 + 2.519 × ln(age) ± 0.146), respectively [4]. In vertebra T6, the neural ossification centers increased in length following the functions: *y* = −15.188 + 6.332 × ln(age) ± 0.629 on the right side and *y* = −15.991 + 6.600 × ln (age) ± 0.629 on the left side. In turn, an increase in width on the right and left sides followed the functions: *y* = −6.716 + 2.814 × ln(age) ± 0.362 and *y* = −7.058 + 2.976 × ln(age) ± 0.323, respectively [28]. The neural ossification center of vertebra L3 on the right and left sides increased in length, following the functions: *y* = − 18.386 + 7.246 × ln(age) ± 0.585 and *y* = − 23.171 + 8.766 × ln(age) ± 0.753, respectively. Their width followed the functions: *y* = − 5.014 + 2.176 × ln(age) ± 0.218 and *y* = −5.624 + 2.343 × ln (age) ± 0.197), respectively [29].

As demonstrated in the current study, the cross-sectional area of the neural ossification centers increased as follows: *y* = −7.362 + 0.780 × age ± 1.700 for the atlas, and *y* = −9.930 + 0.869 × age ± 1.911 for the axis. Of note, a commensurate increase in cross-sectional area of the ossification centers of the right and left neural processes was also shown for vertebra C4: *y* = −9.188 + 0.856 × age ± 2.174 and *y* = −7.570 + 0.768 × age ± 2.200, respectively [4], for vertebra T6: *y* = −5.665 + 0.591 × age ± 1.251 and *y* = −11.281 + 0.853 × age ± 1.653, respectively [28], and for vertebra L3: *y* = − 12.122 + 0.847 × age ± 1.351 and *y* = −11.828 + 0.798 × age ± 1.336, respectively [29]. Our study revealed that the volume of the neural ossification centers in the atlas and axis increased in a commensurate manner. In the atlas, this parameter increased following the function: *y* = −6.417 + 0.836 × age ± 1.924, while in the axis like *y* = −11.592 + 1.087 × age ± 2.509 (*R* ^2^ = 0.71). It should be emphasized that the linear increase in volume of the ossification centers in the right and left neural processes also concerned vertebra C4: *y* = −13.802 + 1.222 × age ± 1.872 and *y* = −11.038 + 1.061 × age ± 1.964, respectively [4], vertebra T6: *y* = −9.279 + 0.849 × age ± 2.302 and *y* = −16.117 + 1.155 × age ± 1.832, respectively [28], and vertebra L3: *y* = −10.902 + 0.854 × age ± 2.141 and *y* = −13.205 + 0.936 × age ± 1.603, respectively [29].

It is noteworthy that our numerical findings obtained from CT and digital image analysis enable to precisely determine the size of the neural ossification centers in the atlas and axis at varying fetal ages, and so may be considered age-specific reference values. This may be extremely expedient when ultrasonically monitoring normal fetal growth and screening for innate faults in fetuses suffering from segmental anomalies of the spine. Nowadays, to diagnose in utero fetuses, the following three methods: 3D-ultrasound, MRI and CT may be involved. Noticeably, the primary method is 3-D ultrasonography which allows to evaluate spinal motion and curvature in real time [10]. The spinal ossification centers at least 1 mm in diameter can be ultrasonically visualized since week 13 onwards [10, 20]. When 3-D ultrasonography offers uncertain results, then superior contrast resolution achieved by MRI may endow us with indispensable information about fetal abnormalities [33]. To minimize the exposure of the fetus to radiation, CT should be held in reserve with relation to specific skeletal malformations, i.e., bone dysplasias that may be equivocal while diagnosed by ultrasound [10, 33].

During both phylogeny and ontogeny cervical vertebrae undergo intense transformation, therefore, they are extremely variable [22, 32, 34]. In neonates, the atlantal anterior arch is usually cartilaginous, but in 20 % of cases, it has no ossification centers. Furthermore, the process of its ossification occurs in the child as late as at the age of 6–24 months [11]. Junewick et al. [11] confirmed that one ossification center usually occurred within the anterior arch. Having excluded cases lacking ossification centers within the atlantal anterior arch, more than one ossification center was found in 27 % of children. Most frequently, two ossification centers were present in 18 % of cases, followed by three ones in 5 % of cases and four ones in 4 % of cases. The variability in the number of ossification centers in the posterior arch is less common than in the anterior arch of the atlas. Karwacki and Schneider [12] studied the ossification process of the first and second cervical vertebrae in children, with particular focusing on the development of the atlantal anterior arch. They observed variability in the number of ossification centers in the atlantal anterior arch, with two centers occurring in 34 % of cases. In turn, a lack of ossification centers was observed in 16.5 % of cases, in which the ossification of the anterior arch progressed medially from the atlantal lateral massae. The authors correlated the differences in the number of ossification centers with the blood supply to that area and the pressure exerted on the anterior arch by the anterior longitudinal ligament. In the present study, no ossification center was observed in the anterior arch of the atlas. Lustrin et al. [16] demonstrated that the two atlantal neural processes fuse at the age of approximately 3 years, and this condition may be often confused with vertebral fracture. The fusion of the anterior and posterior arches of the atlas occurs as late as at the age of approximately 7 years. As far as the axis is concerned, Piatt and Grissom [23] reported that even the five axial ossification centers are present in children: one in either neural process, one located centrally in its body and two in the dens, referring to its apical and central regions. These authors claimed that the axial dens was formed by two individual ossification centers that fused at the age of 7 months of gestation. The axial body fuses with the dens between the age of 3 and 6 years, but the fusion site may be visible until the age of 11 years, and so misinterpreted as vertebral fracture. In the apical region of the dens, a secondary ossification center appears between the age of 3 and 6 years, and fuses completely around the age of 12 years, while the neural processes fuse at the age of approximately 2–3 years [16, 23].

Developmental disorders of the atlas and axis may give symptoms, such as a headache, vertigo, tinnitus, paresis or paralysis [13, 18, 19, 34]. Anomalies of cervical vertebrae are often accompanied by heart defects [34]. The most common group of developmental defects of the spine is dysraphic disorders, exemplified by spina bifida as a result of incomplete closure of the opposite neural processes. Dysraphia of the posterior atlantal arch occurs frequently in otopalatodigital syndrome and the Arnold–Chiari malformation [17, 18]. The unclosed posterior arch of the atlas is observed in 0.4–6.9 % of cases [7, 34], and a lack of the arch is observed sporadically [24]. A congenital loss or reduction of the anterior arch of the atlas is a relatively rare developmental defect, and in radiological diagnostic procedures, it is often confused with fracture, especially when the examination is conducted in a traumatic patient [17]. Among the developmental defects that may consequently lead to disturbances in the cerebrospinal fluid circulation are atlanto-occipital assimilation and occipitalization of the atlas.

In summary, we believe that our factual numerical data concerning neural ossification centers of the atlas and axis in autopsy fetuses may be directly adapted to in utero fetuses with aforementioned spinal abnormalities relating to the neural processes of the first two cervical vertebrae.

## Conclusions

1. The size of neural ossification centers of the atlas and axis shows neither sexual nor bilateral differences.
2. The neural ossification centers of the atlas and axis grow logarithmically in both length and width, and linearly in both cross-sectional area and volume.
3. The numerical data relating to the size of neural ossification centers of the atlas and axis derived from CT and digital-image analysis are considered specific-age reference values of potential relevance in both the ultrasound monitoring and the early detection of spinal abnormalities relating to the neural processes of the first two cervical vertebrae in the fetus.
